# Enhancement of protein thermostability by three consecutive mutations using loop-walking method and machine learning

**DOI:** 10.1038/s41598-021-91339-4

**Published:** 2021-06-04

**Authors:** Kazunori Yoshida, Shun Kawai, Masaya Fujitani, Satoshi Koikeda, Ryuji Kato, Tadashi Ema

**Affiliations:** 1 Innovation Center, Amano Enzyme Inc., Technoplaza, Kakamigahara, Gifu 509-0109 Japan; 2 Division of Applied Chemistry, Graduate School of Natural Science and Technology, Okayama University, Tsushima, Okayama 700-8530 Japan; 3 Department of Basic Medicinal Sciences, Graduate School of Pharmaceutical Sciences, Nagoya University, Nagoya, 464-8601 Japan

**Keywords:** Computational biology and bioinformatics, Machine learning, Biochemistry, Enzymes, Hydrolases

## Abstract

We developed a method to improve protein thermostability, “loop-walking method”. Three consecutive positions in 12 loops of *Burkholderia cepacia* lipase were subjected to random mutagenesis to make 12 libraries. Screening allowed us to identify L7 as a hot-spot loop having an impact on thermostability, and the P233G/L234E/V235M mutant was found from 214 variants in the L7 library. Although a more excellent mutant might be discovered by screening all the 8000 P233X/L234X/V235X mutants, it was difficult to assay all of them. We therefore employed machine learning. Using thermostability data of the 214 mutants, a computational discrimination model was constructed to predict thermostability potentials. Among 7786 combinations ranked in silico, 20 promising candidates were selected and assayed. The P233D/L234P/V235S mutant retained 66% activity after heat treatment at 60 °C for 30 min, which was higher than those of the wild-type enzyme (5%) and the P233G/L234E/V235M mutant (35%).

## Introduction

Enzymes play pivotal roles in various industries, exerting powerful and specific catalytic performances. The inherent enzymatic properties such as catalytic activity, substrate specificity, and optimal temperature are however unsatisfactory in some cases. Enzymatic functions can be strengthened by various methods including protein engineering^1–10^. For example, random mutagenesis^11–18^, rational alteration^19–26^, loop-structure modification^27,28^, and amino-acid sequence alignment^29^ have been studied. Although directed evolution with random mutagenesis is a powerful method^1–10^, both a huge mutant library and a high-throughput screening system are needed to create and select an excellent mutant. To this end, cell-surface display systems^30,31^, flow cytometry^32^, and robotics^33^ have also been developed although costs are required.

Enzymes are often sensitive to temperature and suffer from denaturation. Therefore, various methods have been developed to create thermostable mutants. Loop structures are susceptible to temperature, pH, and solvent, and frequently show high B-factors; the B factor is a crystallographic temperature factor, which can be used as an index for predicting destabilization sites. B-FIT is a method that combines the B-factor with directed evolution, and the thermostability of *Bacillus subtilis* lipase has been improved^34,35^. Directed evolution, DNA shuffling, and yeast cell surface display are also effective for gaining thermostable variants^36–38^. Bioinformatic approaches such as machine learning have also been reported, where a target mutant is designed by analyzing the characteristics of available mutants^39^. A thermostable mutant of *Bacillus subtilis* lipase has been created by using quantitative structure–thermostability relationship models and nonlinear support vector machine^40^. A convolution neural network-based prediction model has been used to create a thermostable mutant of *Rhizomucor miehei* lipase^41^.

**Figure 1:**
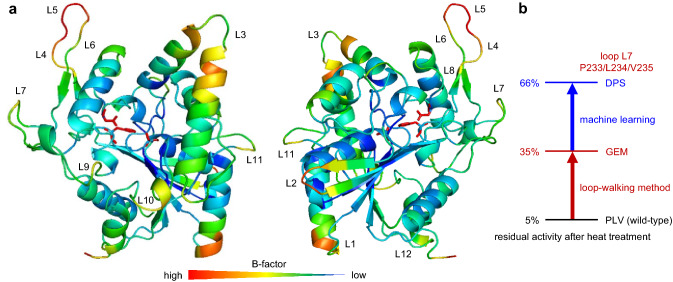
(**a**) Front and back views of LPS (PDB: 1OIL) with the B-factor, where the catalytic triad (S87/H286/D264) is shown in red. Twelve loop regions are indicated: L1_A74/A75/T76, L2_V199/G200/G201, L3_L127/A128/Y129, L4_P216/T217/I218, L5_S219/V220/F221, L6_G222/V223/T224, L7_P233/L234/V235, L8_R258/G259/S260, L9_Q292/L293/L294, L10_G25/V26/L27, L11_P58/N59/G60, L12_Q39/R40/G41. (**b**) A concise summary of thermostability enhancement achieved in this work.

Lipases are enzymes widely used in academia and industry^42,43^. *Burkholderia cepacia* lipase, commercialized as lipase PS (LPS), is one of the most useful biocatalysts, and robust mutants are required. Here we have developed a “loop-walking method” for the creation of thermostable mutants. We introduced random mutations into three consecutive positions in each of twelve loop regions of LPS (L1 to L12, Fig. 1), expecting the synergistic effect of the contiguous triple mutations. Screening of twelve mutant libraries allowed us to identify L7 as a hot-spot loop having an impact on thermostability, and the P233G/L234E/V235M mutant was found. Because this triple mutant was found by the screening of 214 variants in the L7 library, a more excellent mutant might be discovered by the screening of all the 8000 (= 20^3^) possible P233X/L234X/V235X mutants. However, it was experimentally difficult to cover all of them. Therefore, we introduced machine learning to effectively narrow down the possible combinations, based on the concept of our in silico mutant screening, which analyzes physicochemical rules in the experimental data with multivariate analysis^44–55^. By modeling the thermostability data of the 214 variants, all the remaining triple combinations were ranked in silico. Top 20 candidates were experimentally prepared, and the P233D/L234G/V235G and P233D/L234P/V235S triple mutants were discovered. The loop-walking method in combination of machine learning is a powerful strategy for the creation of thermostable mutants of proteins.

## Results and discussion

### Exploration of triple mutants with the loop-walking method

**Figure 2:**
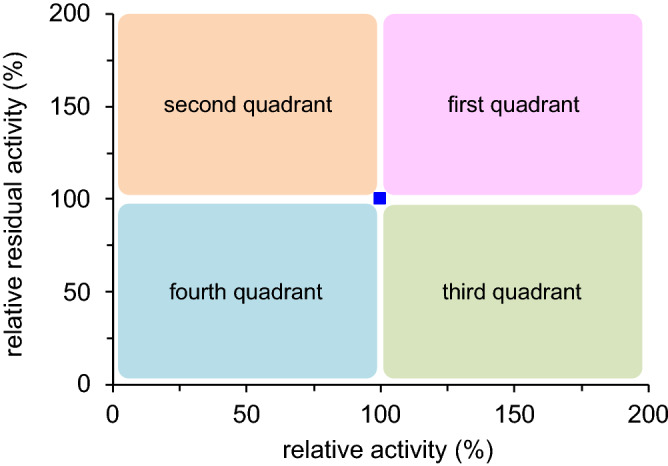
Quadrant classification of mutants: relative activity without heat treatment (horizontal axis) and relative residual activity after heat treatment at 60 °C for 30 min (vertical axis) as compared to the wild-type enzyme (blue square).

Using the crystal structure of LPS (PDB code: 1OIL)^56^, we selected twelve loop regions (L1 to L12, Fig. 1) and introduced random mutations into three consecutive positions to make twelve mutant libraries. Approximately 200 variants for each library were picked up and produced by recombinant *Escherichia coli* (*E. coli*)^57^, and enzymatic activity and residual activity after heat treatment (60 °C for 30 min) were measured. The relative activity and residual activity of mutants as compared to those of the wild-type enzyme are visualized by quadrant classification (Fig. 2). The mutants with improved thermostability appear in the first and second quadrants, while the mutants with reduced thermostability appear in the third and fourth quadrants. The difference between the first and second quadrants or between the third and fourth quadrants represents the difference in enzymatic activity without heat treatment. Therefore, an ideal variant with improved activity and thermostability will appear in the first quadrant.

**Figure 3:**
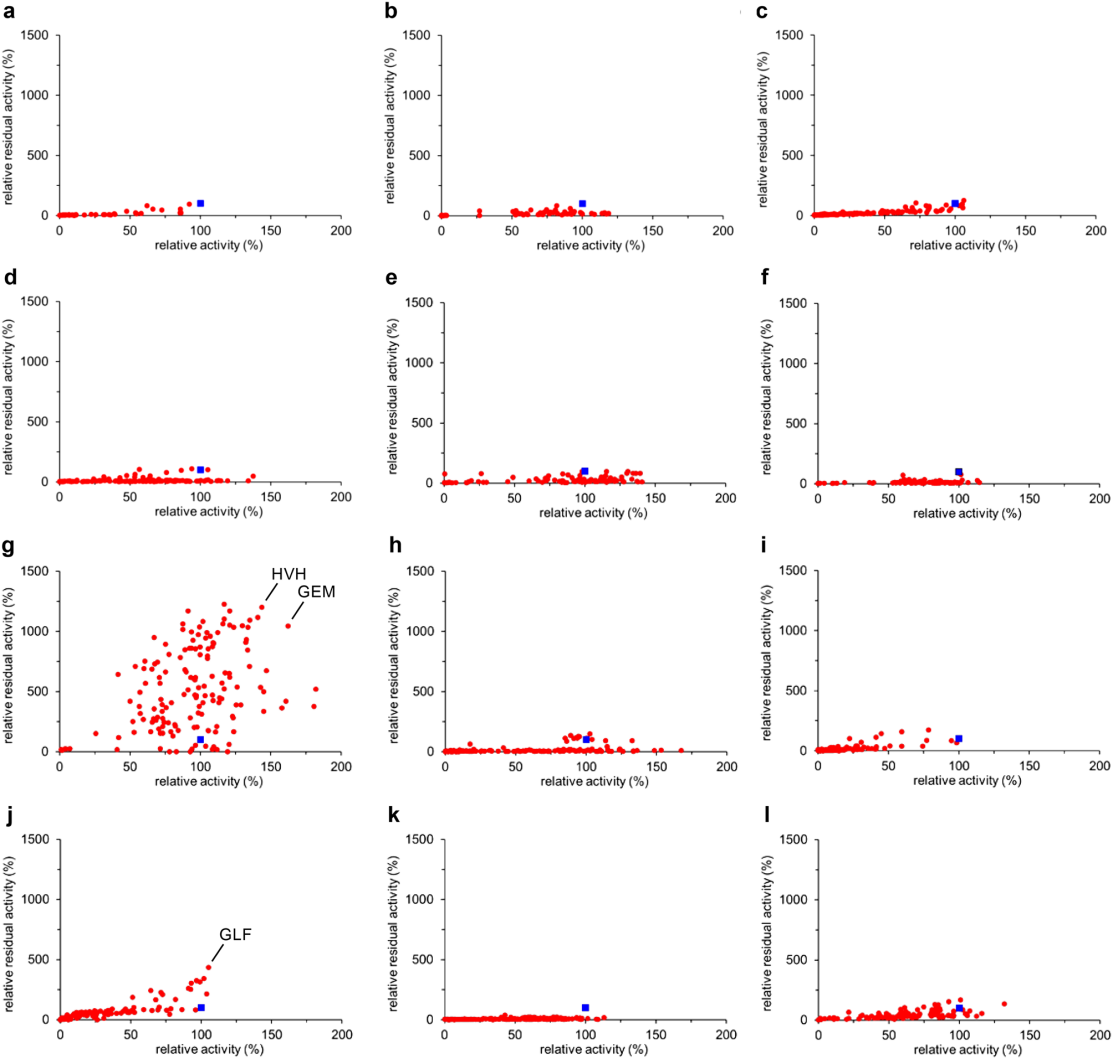
Thermostability plots for the twelve libraries with random mutations in each loop region: (**a**) L1, (**b**) L2, (**c**) L3, (**d**) L4, (**e**) L5, (**f**) L6, (**g**) L7, (**h**) L8, (**i**) L9, (**j**) L10, (**k**) L11, and (**l**) L12. Relative activity without heat treatment (horizontal axis) and relative residual activity after heat treatment at 60 °C for 30 min (vertical axis) are based on the wild-type enzyme (blue square).

Figure 3 shows the results of the assay. To our delight, many variants having mutations in the L7 region appeared mainly in the first or second quadrant (Fig. 3g). The P233G/L234E/V235M and P233H/L234V/V235H mutants were the best ones, showing 11-fold and 12-fold residual activity, respectively, as compared with the wild-type enzyme. Obviously, L7 is a hot-spot loop capable of enhancing thermostability. In sharp contrast, all the remaining libraries had most data in the third and fourth quadrants although the L10 library seemed to be slightly promising. Interestingly, no positive variants were obtained in the L2 and L5 libraries (Fig. 3b,e) despite the high B-factors around the L2 and L5 regions (Fig. 1). This result sharply contrasts with the previous reports, where loop regions with high B-factors were altered to create excellent variants of various enzymes^58^, including *Bacillus subtilis* lipase^34,35^. The loop-walking method has good potential for the creation of thermostable mutants that cannot be obtained by the B-FIT method, which always depends on the B-factors of X-ray crystal structures.

The two best triple mutants were compared in more detail. As a result of heat treatment at 70 °C for 30 min, the P233G/L234E/V235M mutant was more thermostable than the P233H/L234V/V235H mutant (Fig. S1a,c); the former exhibited a residual activity of more than 40% whereas the latter showed little or no residual activity. On the other hand, although the P233G/L234E/V235M mutant showed lower activity at 60 °C than the P233H/L234V/V235H mutant, they exhibited comparable activities at 70 °C (Fig. S1b,d). Based on these results, the P233G/L234E/V235M mutant was taken as the best one.

### Synergistic effect of triple mutations

**Figure 4:**
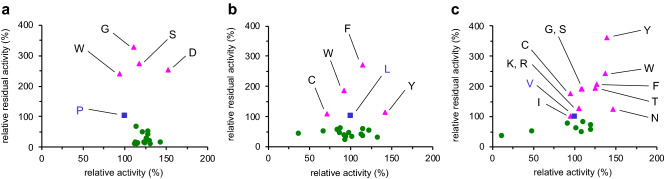
Thermostability plots for (**a**) P233X, (**b**) L234X, and (**c**) V235X single mutants using relative activity without heat treatment (horizontal axis) and relative residual activity after heat treatment at 60 °C for 30 min (vertical axis). The blue square represents the wild-type enzyme while the pink triangle represents the mutants with improved thermostability, and the green circle represents the mutants with reduced thermostability.

It is interesting to investigate the synergistic effect of the triple mutations. New libraries of saturation mutagenesis were constructed for each amino-acid residue (P233, L234, and V235), and enzymatic activity and residual activity after heat treatment were measured (Fig. 4). Several single mutants (P233D/G/S/W, L234C/F/W/Y, V235C/F/G/I/K/N/R/S/T/W/Y) showed improved thermostability (residual activity), among which the P233D/G/S, L234F/Y, and V235F/G/K/N/R/S/T/W/Y mutants showed improved enzymatic activity as well. However, these single mutants were inferior to the best triple mutant, which suggests the synergetic effect of the three amino-acid residues of the triple mutant (Figs. 3g, 4). Furthermore, the high thermostability of the P233G/L234E/V235M and P233H/L234V/V235H mutants is difficult to rationalize with Fig. 4; for example, single mutations such as P233H, L234E/V, and V235M/H resulted in no enhancement of thermostability (residual activity): P233G = 327%, L234E = 45%, V235M = 62%; P233H = 20%, L234V = 59%, V235H = 36%. Obviously, the effect of the triple mutations on thermostability (Fig. 3g) is much greater than the sum of the individual effects. This fact strongly supports the synergetic effect of the three consecutive amino-acid residues introduced by the loop-walking method. The synergistic effect of the three amino-acid residues of the best mutant in the L10 library, G25G/V26L/L27F, was also confirmed in the same way (Fig. 3j, Supplemental Fig. S2).

### Prediction of promising mutants by machine learning

Because L7 was identified as a hot-spot loop by the screening of 214 variants, we expected that a more excellent variant might be discovered by the comprehensive examination of all the 8000 (= 20^3^) amino-acid combinations in the L7 library. To accelerate our exploration, we decided to employ machine learning (multivariate analysis) with the data of the 214 mutants. The amino-acid residue in each position was individually converted into 13 physicochemical parameters (Fig. S3)^44–55^ as explanatory valuables and trained with their thermostability activities as objective variables. In this model construction step, the total data were divided into two categories, “improved” or “non-improved”, and a discrimination model for reducing non-effective combinations was constructed. Since the model accuracy was high (94.5%), 7786 amino-acid combinations, which are the remaining combination candidates in the 8000 combinations, were evaluated in silico. From this in silico screening, 5292 combination candidates were predicted to be improved. To select more reliable combination candidates, we constructed the second discrimination model that can classify “high thermostability improvement” and “medium thermostability improvement” (model accuracy of 85.5%). With this model, we evaluated 5292 combination candidates in silico and ranked them with their prediction possibilities (Tables S8, S9).

### Experimental validation of the prediction model

**Figure 5:**
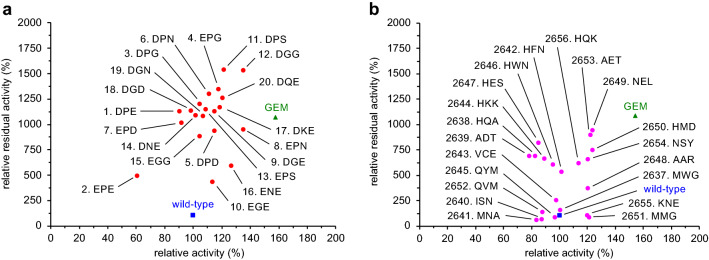
Thermostability plots for (**a**) high 20 mutants and (**b**) medium 20 mutants. Relative activity without heat treatment (horizontal axis) and relative residual activity after heat treatment at 60 °C for 30 min (vertical axis) are based on the wild-type enzyme (blue square). The predicted ranking number is indicated together with three amino-acid residues in the L7 region.

To confirm the thermostability of the predicted candidates (Tables S8, S9), we experimentally prepared 40 mutants: 20 mutants predicted to show “high thermostability improvement” (high 20 mutants) and 20 mutants predicted to show “medium thermostability improvement” (medium 20 mutants) (Fig. 5). As a result of experiments, all the high 20 mutants were more thermostable than the wild-type enzyme, some of which exhibited thermostability that was higher than 1000% with a hit rate of 70% (14 out of 20) (Fig. 5a). In addition, most of the mutants exhibited improved enzymatic activity (first quadrant), and the hit rate reached 80% (16 out of 20). This hit rate was much higher than the original hit rate in the first screening (50%, 108 out of 214). To our delight, two top mutants, P233D/L234G/V235G and P233D/L234P/V235S (relative residual activity: 1500%), were clearly superior to the P233G/L234E/V235M mutant (relative residual activity: 1100%). The representative raw data are shown in Table 1. Although the residual activity of the wild-type enzyme decreased to 5% after heat treatment at 60 °C for 30 min, the corresponding value for the P233G/L234E/V235M mutant was 35%, and P233D/L234G/V235G and P233D/L234P/V235S mutants retained 59% and 66% activity, respectively, after the heat treatment. In addition, these variants were more active without heat treatment than the wild-type enzyme. On the other hand, although most of the medium 20 mutants showed higher thermostability than the wild-type enzyme, the improvement level was modest (< 1000%) (Fig. 5b). Overall, our prediction model is reliable, successfully extracting rules for the improvement of thermostability from the limited number of the first screening data.

**Table 1:** Raw data of enzymatic activity and residual activity.

Lipase	Enzymatic activity without heat treatment (U/mL)	Residual activity after heat treatment at 60 °C for 30 min (U/mL)
Wild-type	1000	52 (5%)
P233G/L234E/V235M	1580	560 (35%)
P233D/L234G/V235G	1350	800 (59%)
P233D/L234P/V235S	1220	800 (66%)

### Finding rules in the L7 region

It is significant to find a rule for acquiring protein thermostability. The careful inspection of the predicted amino-acid combinations (Fig. 5a, Table S8) and the weighted parameters for the prediction of high/medium improvement (Table 2, Fig. S3) allowed us to discover rules of amino-acid combinations. First of all, position 233 is the most weighted and influential. Although “polarity” (high in Arg, Lys, His, Asp, and Glu) has a positive weight (0.129), “isoelectric point” (high in Arg and Lys) has a negative impact (− 0.27). In addition, this position disfavors aromatic residues; “side-chain contribution to protein stability” (high in Phe and Trp) and “free energy in beta-strand region” (high in Pro and Gly) have negative weights (− 0.348 and − 0.196, respectively). Consequently, acidic residues (Asp or Glu) make major positive contributions. On the other hand, position 234 is less influential, exhibiting small weight values. Nevertheless, there are some amino-acid preferences; “side chain interaction parameter” (high in Lys, Pro, Gln, Glu, and Asp) and “free energy in beta-strand region” (high in Pro and Gly) are positively weighted (0.084 and 0.047, respectively) whereas “polarity” (high in Arg, Lys, His, Asp, and Glu) is negatively weighted (− 0.041). Position 235 disfavors amino acids with a bulky side chain; “the stability scale from the knowledge-based atom–atom potential” (high in Phe, Trp, and Tyr) is negatively weighted (− 0.219). In contrast, “free energy in beta-strand region” (high in Pro and Gly) is positively weighted (0.043). Accordingly, the Pro and Gly residues at positions 234 and 235 are likely to have a positive effect on thermostability. Overall, the two top mutants, P233D/L234G/V235G and P233D/L234P/V235S, are consistent with the above rules.

**Table 2:** Physicochemical parameters weighted in the discrimination model for mutants showing high/medium thermostability.

Physicochemical parameter	Weight in the model		
	233	234	235
Isoelectric point^45^	− 0.270	− 0.002	0
Normalized van der Waals volume^46^	− 0.102	0	0
Alpha-helix indices for beta-proteins^47^	0	0	0
Beta-strand indices for beta-proteins^47^	− 0.066	0	− 0.100
Side-chain contribution to protein stability^48^	− 0.348	0	0
The stability scale from knowledge-based atom–atom potential^49^	0	0	− 0.219
Hydropathy index^50^	0	0	− 0.027
Normalized frequency of turn^51^	0.023	0	0
Free energy in beta-strand region^52^	− 0.196	0.047	0.043
Free energy in alpha-helical region^52^	0	0	0
Polarity^45^	0.129	− 0.041	0
Side chain interaction parameter^53^	0	0.084	0
Amino acid distribution^54^	0	0.013	0

Positive values indicate parameter contribution to “high thermostability” while negative values indicate parameter contribution to “medium thermostability”.

**Figure 6:**
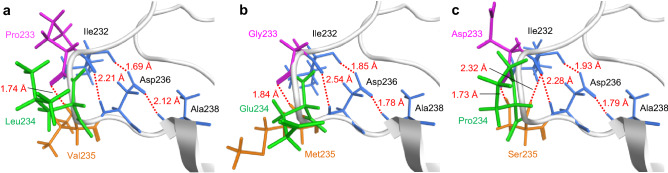
The L7 loop region of (**a**) the wild-type enzyme (PDB: 1OIL), (**b**) P233G/L234E/V235M, and (**c**) P233D/L234P/V235S.

The result that the P233D/L234P/V235S triple mutant exerted the most excellent thermostability was surprising because the L234P single mutant exhibited no enhanced thermostability (Fig. 4b, residual activity 34%). This fact supports the synergy effect of the three consecutive mutations, which is one of the most important advantages of the loop-walking method over conventional random mutagenesis. To gain a molecular insight into the origin of heat resistance enhanced by these amino-acid substitutions, three-dimensional structural models were constructed (Fig. 6). The wild-type enzyme and the P233G/L234E/V235M triple mutant have a hydrogen bond between the backbone amide groups of residues 233 and 235 (Fig. 6a,b), while the P233D/L234P/V235S triple mutant has hydrogen bonds between the protein backbone C = O group of Asp233 and the sidechain OH group of Ser235 and between the backbone amide groups of residues 232 and 235, retaining the hydrogen-bonding networks between Ile232, Asp236, and Ala238 (Fig. 6c). These attractive interactions are likely to rigidify the loop, contributing to the high thermostability of the whole protein.

## Conclusion

Robust mutants are necessary for finding more applications in academia and industry^59–61^. Here we have developed the loop-walking method for the enhancement of protein thermostability; random mutations are introduced into three consecutive amino-acid residues of each loop, and mutants with high thermostability are searched for. Using this method, we have successfully improved the thermostability of *Burkholderia cepacia* lipase, lipase PS (LPS). The twelve loop regions (L1 to L12) were genetically modified to make twelve mutant libraries, from which the P233G/L234E/V235M mutant (relative residual activity: 1100%) was discovered. Although the residual activity of the wild-type enzyme decreased to 5% after heat treatment at 60 °C for 30 min, that of the P233G/L234E/V235M mutant was 35%. Importantly, we have confirmed the synergistic effect of the three consecutive mutations on the thermostability of the triple mutant. Although L7 was identified as a hot-spot loop by the screening of 214 variants, it was difficult to assay all the 8000 (= 20^3^) combinations. To enhance the efficiency of mutant screening, we introduced machine learning (multivariate analysis). Using mutation data linked with experimentally determined performances of the 214 mutants as training data, we predicted promising mutants with improved thermostability. As a result of experiments, the P233D/L234G/V235G and P233D/L234P/V235S mutants (relative residual activity: 1500%) were discovered; the latter mutant retained 66% activity after heat treatment at 60 °C for 30 min, which was much higher than that of the wild-type enzyme (5%). We studied physicochemical rules from the weighted parameters of each amino acid predicted by the machine learning model. We have noticed rules of thermostability improvement, illuminating the mechanistic aspect. Some of the triple mutants obtained in this study are promising biocatalysts, and the loop-walking method combined with machine learning is a powerful strategy, which will be useful for the optimization of various biocatalysts in future.

## Methods

### General methods

Takara PCR Thermal Cycler Dice Gradient was used for DNA amplifications. PrimeSTAR GXL DNA polymerase (Takara Bio Inc., 1 μL), 5× PrimeSTAR GXL buffer (10 μL), dNTP Mixture (2.5 mM each) (4 μL), forward primer (10 pmol), reverse primer (10 pmol), and template (10 ng) were used after filling up to 50 μL with sterilized distilled water. PCR was done for 15 cycles of (98 °C for 10 s, 60 °C for 30 s, and 68 °C for 1.5 min). DNA manipulation reagents such as restriction enzymes and ligases were purchased from Takara Bio Inc. and TOYOBO. To confirm nucleotide sequences, pET upstream primer (5′-ATGCGTCCGGCGTAGA-3′), duetdown1 primer (5′-GATTATGCGGCCGTGTACAA-3′), duetup2 primer (5′-TTGTACACGGCCGCATAATC-3′), T7 terminator primer (5′-GCTAGTTATTGCTCAGCGG-3′) were used.

### Preparation of the *E. coli* codon-optimized LPS gene

*Burkholderia cepacia* lipase was produced with the structural gene (LipA) and the chaperone gene (LipX); the former is a lipase-encoding gene, and the latter is a private chaperone responsible for the folding of the lipase. For the recombinant *E. coli* expression of LPS, codon-optimized structural gene (LPS_LipA_opti) and chaperone gene (LPS_LipX_opti) were prepared by artificial gene synthesis (GenScript).

### Preparation of the recombinant *E. coli* expression plasmid

The LPS *E. coli* expression plasmid was constructed by referring to the reference^57^. The DNA fragment with linker sequences (*Nco* I, *Hin*d III) was obtained by PCR amplification using primers (forward: 5′-TTTTCCATGGCTCGTTCTATGCGTTCTCG-3′, reverse: 5′-AAAAAAGCTTAAACACCCGCCAGTTTCAGACGG-3′) and the synthetic structural gene (LPS_LipA_opti), where restriction sites for *Nco* I and *Hin*d III are underlined. The PCR product was electrophoresed on 1% agarose gel to cut out a target band and purified with NucleoSpin DNA clean-up kit (QIAGEN). The purified DNA fragment and expression vector (pETDuet-1) were digested with *Nco* I and *Hin*d III, and both fragments were ligated using DNA Ligation Kit < Mighty Mix >. To obtain the recombinant strain (*E. coli* LPS_LipA), the plasmid obtained was used for the transformation of *E. coli* DH5α by the heat-shock method. The *E. coli* LPS_LipA strain was inoculated into a liquid medium (1 mL L broth (Invitrogen) with 100 μg/mL ampicillin per test tube) and cultured at 37 °C and 140 rpm for 16 h. The expression plasmid (pETLPS_LipA) was extracted from the culture broth using Nucleospin plasmid easypure kit (Macherey nagel). The chaperone gene (LPS_LipX_opti) was inserted into the expression plasmid (pETLPS_LipA). The DNA fragment with linker sequences (*Nde* I, *Xho* I) was obtained by PCR amplification using primers (forward: 5′-TTTTCATATGACCGCACGTGAAGGTCGCGC-3′, reverse: 5′-AAAACTCGAGTTACTGTGCAGAACCCGCACCG-3′) and the synthetic structural gene (LPS_LipX_opti), where the restriction sites for *Nde* I and *Xho* I are underlined. The PCR product was electrophoresed on 1% agarose gel to cut out a target band and purified. The purified DNA fragment and expression vector (pETLPS_LipA) were digested with *Nde* I and *Xho* I, and both fragments were ligated using DNA Ligation Kit < Mighty Mix >. To obtain a recombinant strain (*E. coli* LPS_LipA/LipX), the plasmid obtained was used for the transformation of *E. coli* DH5α by the heat-shock method. The *E. coli* LPS_LipA/LipX strain was inoculated into a liquid medium (1 mL L broth with 100 μg/mL ampicillin per test tube) and cultured at 37 °C and 140 rpm for 16 h. The expression plasmid (pETLPS_LipA/LipX) was extracted from culture broth with Nucleospin plasmid easypure kit.

### Preparation of the recombinant *E. coli* expression strain

The expression plasmid (pETLPS_LipA/LipX) was used for the transformation of *E. coli* BL21(DE3) to obtain the recombinant *E. coli* expression strain (*E. coli* LPS_LipA/LipX).

### Preparation of the random mutant strain of each mutation region

The random mutation primers were designed to prepare a random mutation library for each loop region. The PCR product was obtained by PCR amplification using each designed primer (Table S1) and the LPS expression plasmid (pETLPS_LipA/LipX). The PCR products were digested with *Dpn* I, and the digested PCR products were ligated with T4 Polynucleotide Kinase and Ligation high Ver.2 (TOYOBO). To obtain the LPS random mutant expression strain for each mutation loop region (*E. coli* LPS_Ran_L1 to LPS_Ran_L12), the ligated plasmid was used for the transformation of *E. coli* BL21(DE3).

### Preparation of the random mutation library

The random mutation library was prepared from the LPS random mutants (*E. coli* LPS_Ran_L1 to LPS_Ran_L12) in two steps. The first step is the selection of mutants with hydrolytic activity. Each random mutant strain was spread onto a plate medium (LB agar plate with 100 μg/mL ampicillin, 0.1% tributyrin) and cultivated at 37 °C for 24 h, and a mutant strain forming a clear halo was selected. The second step is the preparation of the enzyme extract from the selected mutant strain. The selected mutant strains were inoculated into a liquid medium (1 mL terrific broth with 100 μg/mL ampicillin) in 96 deep-well plate (Coastar) and cultured at 33 °C and 1000 rpm for 48 h with a plate shaker (TAITEC), during which 0.1 mM IPTG was added to induce the enzyme expression at 24 h. The cell pellet was collected from the culture broth by centrifugation (3300×*g* × 15 min, 4 °C). To extract the enzyme from the cell pellet, a lysing agent (1 mL B-PER (Thermo Fisher Scientific)) was added and incubated at 25 °C and 1000 rpm for 2 h using a plate shaker. The lysis supernatant was collected by centrifugation (3300×*g* × 15 min, 4 °C).

### Preparation of the site-saturation mutagenesis library

To prepare the site-saturation mutagenesis library for each mutation site (G25, V26, L27, P233, L234, and V235), primers were designed as shown in Tables S2–S7. The mutation was performed by PCR amplification using the designed primers and pETLPS_LipA/LipX. The PCR product was digested with *Dpn* I at 37 °C for 16 h, and the digested PCR product was ligated with T4 Polynucleotide Kinase and Ligation high Ver.2. The ligated PCR product was used for the transformation of *E. coli* BL21(DE3) to construct each variant expression strain (*E. coli* LPS_G25A to LPS_G25Y, *E. coli* LPS_V26A to LPS_V26Y, *E. coli* LPS_L27A to LPS_L27Y, *E. coli* LPS_P233A to LPS_P233Y, *E. coli* LPS_L234A to LPS_L234Y, and *E. coli* LPS_V235A to LPS_V235Y). Each mutation was confirmed by DNA sequencing. Each LPS mutant *E. coli* expression strain was inoculated into a liquid medium (1 mL terrific broth with 100 μg/mL ampicillin in 96 deep-well plate (Greiner)) and cultured at 33 °C and 1000 rpm for 48 h with a plate shaker, during which 0.1 mM IPTG was added to induce the enzyme expression at 24 h. The cell pellet was collected from the culture broth by centrifugation (3300×*g* × 15 min, 4 °C). To extract the enzyme from the cell pellet, a lysing agent (1 mL B-PER) was added and incubated at 25 °C and 1000 rpm for 2 h with a plate shaker. The lysis supernatant was collected by centrifugation (3300×*g* × 15 min, 4 °C).

### Evaluation of thermostability of mutants

The thermostability of the wild-type enzyme or variant was evaluated by the residual activity of the sample after heat treatment, for example, at 60 °C for 30 min. The lipase activity was determined by using Lipase Kit S (DS Pharma Biomedical) according to the standard manual in the kit, and the absorbance at 412 nm was measured on a PowerScanHT (DS Pharma Biomedical). One enzyme unit was defined as the amount of enzyme hydrolyzing 1 μmol of 2,3-dimercaptopropan-1-ol tributyrate (BALB) per minute under the assay conditions, which was detected by the yellow color of 2-nitro-5-thiobenzoate generated by the addition of 5,5′-dithiobis(2-nitrobenzoic acid) (Ellman’s reagent). Averaged data of three measurements are reported. The experimental errors were less than 15%.

### Thermostability and optimum temperature

The thermostability of mutants was evaluated by comparing the residual activity of the samples that were heat-treated at each temperature (from 40 to 70 °C) for 30 min. The optimum temperature was evaluated by comparing the hydrolytic activity of the sample at each temperature (from 40 to 70 °C). The results are shown in Fig. S1.

### Data processing and model construction for thermostability improvement prediction

The mutant thermostability evaluation data (214 mutants from the first screening) was converted into dataset for machine learning. The mutant profile, the amino-acid usage for each mutant at three positions (P233, L234, and V235), was converted into 13 physicochemical parameters (Table 2) for each position^45–54^. All physicochemical parameters were downloaded from AAindex (Fig. S3) (https://www.genome.jp/aaindex/). Using 544 amino acid indices registered in AAindex (version 9.1, as of January 2008), 21 major clusters with high correlations were selected through hierarchical clustering as representative amino acid parameter clusters. From such clusters, 13 indices with implementable meaning were manually selected. Since they are selected from the unsupervised clustering of total AAindex indices, selected indices serve as objectively selected independent parameters to describe physicochemical properties of amino acids. Isoelectric point, normalized van der Waals volume, and hydropathy index have been used to model lipase enantioselectivity^44^, while isoelectric point, normalized van der Waals volume, side-chain contribution to protein stability, hydropathy index, normalized frequency of turn, polarity, and side chain interaction parameter have been used to model oligopeptide transporter^55^. In this work, amino acid indices were increased for more descriptive performances. Each position of mutation (P233, L234, and V235) was converted into the physicochemical properties described by these 13 indices. Therefore, the final explanatory valuables were 39 parameters (13 parameters × 3 positions). The thermostability activity was calculated as the ratio of the residual activity after heat treatment (60  °C for 30 min) to the enzymatic activity without heat treatment. As a result of our preliminary analysis, the dataset of the thermostability activity furnished a better regression model than the raw dataset of either the residual activity after heat treatment or the enzymatic activity without heat treatment. Therefore, the dataset of the thermostability activity was utilized for further prediction analysis. The thermostability activity was normalized in total sample and categorized into three levels [high (73 data: top 34%), low (73 data: bottom 34%), and medium (72 data: the rest of data)] using their ranking of thermostability improvement. Such data stratification was introduced since a total data modeling resulted in a low accuracy (< 75%). Dividing the dataset into the 3-equal parts successfully enhanced model accuracies (high/low discrimination model: 93.1%, medium/low discrimination model: 93.5%, high + medium/low discrimination model: 94.5%, high/medium discrimination model: 85.5%). For the first discrimination analysis, a discrimination model for “high + medium variants (improved)” vs. “low variants (non-improved)” was constructed to screen the candidates briefly. The bottom 34% variants (low) were labeled as “non-improved”, and the rest of the variants were labeled as “improved” for model training. The discrimination analysis model was constructed by LASSO (least absolute shrinkage and selection operator) regression and validated by leave-one-out cross validation. After the model construction, 7786 remaining amino acid combinations among 8000 total combinations were synthesized in silico and converted into 39 parameters. Such in silico synthesized amino acid combination candidates were applied to the improved/non-improved discrimination model, and predicted “improved” candidates were selected. For the second discrimination analysis, a discrimination model for “high variants (high)” vs. “medium variants (medium)” was constructed. High and medium thermostability improvement data were categorized as “high” and “medium”, and its discrimination analysis model was also constructed by LASSO. Leave-one-out cross validation was used for the evaluation of the constructed model. The 5292 candidates that were predicted as “improved” in the first discrimination model were predicted by the second model. From the second discrimination model, their high/medium discrimination probabilities were calculated for all the candidates and listed as prediction ranking (Tables S8, S9). All calculation and data analysis program was coded by R (https://cran.r-project.org/).

### Preparation and evaluation of 40 mutants selected from ranking predicted mutants

The 40 mutants were selected from the prediction ranking list: 20 mutants predicted to show “high thermostability improvement” (= high 20 mutants) and 20 mutants predicted to show “medium thermostability improvement” (= medium 20 mutants). To create these mutants, each mutation PCR primer was designed (Tables S10, S11). Each mutant was prepared by site-directed mutagenesis using each designed PCR primer and expression plasmid (pETLPS_LipA/LipX) and then transformed into *E. coli* BL21(DE3). Cultivation of each mutant, preparation of enzyme extract, and evaluation of thermostability were carried out as described above.

### Structures of LPS and the triple mutants

The structure of LPS (PDB: 1OIL) was optimized by QuickPrep function of MOE (Molecular Operating Environment, MOLSIS), where Amber 10: EHT was used as a force field. The structures of the triple mutants (P233G/L234E/V235M and P233D/L234P/V235S) were created by using LPS as a template with Protein Design and QuickPrep functions of MOE.

## Supplementary Information

Supplementary Information.
