# Supplementary material for: Enhancement of protein thermostability by three consecutive mutations using loop-walking method and machine learning
Source: Sci Rep. 2021 Jun 4;11:11883. doi: 10.1038/s41598-021-91339-4 (PMC8178419; doi:10.1038/s41598-021-91339-4)
Supplement: Supplementary file 1 — Supplementary Information. [file 41598_2021_91339_MOESM1_ESM.pdf]

Supplementary Information

Enhancement of Protein Thermostability by Three Consecutive Mutations Using Loop-Walking Method and Machine Learning

Kazunori Yoshida,[1][2] Shun Kawai,[3] Masaya Fujitani,[3] Satoshi Koikeda,*[1] Ryuji Kato,*[3] and Tadashi Ema*[2]

[1] Innovation Center, Amano Enzyme Inc., Technoplaza, Kakamigahara, Gifu 509-0109, Japan.
[2] Division of Applied Chemistry, Graduate School of Natural Science and Technology, Okayama University, Tsushima, Okayama 700-8530, Japan.
[3] Department of Basic Medicinal Sciences, Graduate School of Pharmaceutical Sciences, Nagoya University, Nagoya 464-8601, Japan.

Table S1. Random mutation primers for LPS-L1 to LPS-L12.

no.	mutation point	primer	sequence (5' - 3')
1	LPS-L1_A74/A75/T76	forward	NNKNNKNNKGGCGCAACCAAAGTTAACCTGGTTG
		reverse	CAGCACAGTTTTCACATACGCCAGC
2	LPS-L2_V199/G200/G201	forward	NNKNNKNNKAACACTCACCTGCTGTACTCTTGGGC
		reverse	AGTCTCGGTCGGCGCACC
3	LPS-L3_L127/A128/Y129	forward	NNKNNKNNKGACCCGACTGGCCTGTCTTCTACC
		reverse	AACGCCCTGAACGAAATCCGCG
4	LPS-L4_P216/T217/I218	forward	NNKNNKNNKTCTGTTTTCGGTGTTACTGGTGCGAC
		reverse	CTGGATCGCAGTACCCGCCCAAG
5	LPS-L5_S219/V220/F221	forward	NNKNNKNNKGGTGTTACTGGTGCGACTGATACCTCTAC
		reverse	GATGGTCGGCTGGATCGCAGTAC
6	LPS-L6_G222/V223/T224	forward	NNKNNKNNKGGTGCGACTGATACCTCTACTATCCCGC
		reverse	GAAAACAGAGATGGTCGGCTGGATCGC
7	LPS-L7_P233/L234/V235	forward	NNKNNKNNKGATCCGGCAAACGCACTGGACC
		reverse	GATAGTAGAGGTATCAGTCGCACCAGTAAC
8	LPS-L8_R258/G259/S260	forward	NNKNNKNNKGGTCAGAACGATGGTGTGGTGTCTAAGTG
		reverse	GTTCACCATAACGGTGCCGGTACCAAAC
9	LPS-L9_Q292/L293/L294	forward	NNKNNKNNKGGTGTTCGTGGTGCTAACGCGGAAGATC
		reverse	GTTGATCTCGTCCAGGTGGTTCCATTTGTAAGAG
10	LPS-L10_G25/V26/L27	forward	NNKNNKNNKGAGTACTGGTACGGTATTCAGGAAGACCTGC
		reverse	AGCATACTTATCGGTGCCAGTCAGACCATG
11	LPS-L11_P58/N59/G60	forward	NNKNNKNNKCGCGGCGAACAGCTGCTGGCGTATGTGAAAAC
		reverse	GCCGTCGTCGGACTGGAAACCAGACAGGTTC
12	LPS-L12_Q39/R40/G41	forward	NNKNNKNNKGCGACTGTTTACGTTGCGAACCTGTCTGGTTTC
		reverse	CTGCAGGTCTTCCTGAATACCGTACCAGTACTC

Table S2. Mutation primers for LPS_Gly25.

mutation point	amino acid substitution	primer name		sequence (5' - 3')
Gly25	Ala	forward	LPS_G25A-F1	GCGGTTCTGGAGTACTGGTACGGTATTCAGGAAGACC
	Cys		LPS_G25C-F1	TGCGTTCTGGAGTACTGGTACGGTATTCAGGAAGACC
	Asp		LPS_G25D-F1	GATGTTCTGGAGTACTGGTACGGTATTCAGGAAGACC
	Glu		LPS_G25E-F1	GAAGTTCTGGAGTACTGGTACGGTATTCAGGAAGACC
	Phe		LPS_G25F-F1	TTCGTTCTGGAGTACTGGTACGGTATTCAGGAAGACC
	Gly		LPS_G25G-F1	GGCGTTCTGGAGTACTGGTACGGTATTCAGGAAGACC
	His		LPS_G25H-F1	CATGTTCTGGAGTACTGGTACGGTATTCAGGAAGACC
	Ile		LPS_G25I-F1	ATTGTTCTGGAGTACTGGTACGGTATTCAGGAAGACC
	Lys		LPS_G25K-F1	AAAGTTCTGGAGTACTGGTACGGTATTCAGGAAGACC
	Leu		LPS_G25L-F1	CTGGTTCTGGAGTACTGGTACGGTATTCAGGAAGACC
	Met		LPS_G25M-F1	ATGGTTCTGGAGTACTGGTACGGTATTCAGGAAGACC
	Asn		LPS_G25N-F1	AACGTTCTGGAGTACTGGTACGGTATTCAGGAAGACC
	Pro		LPS_G25P-F1	CCGGTTCTGGAGTACTGGTACGGTATTCAGGAAGACC
	Gln		LPS_G25Q-F1	CAGGTTCTGGAGTACTGGTACGGTATTCAGGAAGACC
	Arg		LPS_G25R-F1	CGTGTTCTGGAGTACTGGTACGGTATTCAGGAAGACC
	Ser		LPS_G25S-F1	AGCGTTCTGGAGTACTGGTACGGTATTCAGGAAGACC
	Thr		LPS_G25T-F1	ACCGTTCTGGAGTACTGGTACGGTATTCAGGAAGACC
	Val		LPS_G25V-F1	GTGGTTCTGGAGTACTGGTACGGTATTCAGGAAGACC
	Trp		LPS_G25W-F1	TGGGTTCTGGAGTACTGGTACGGTATTCAGGAAGACC
	Tyr		LPS_G25Y-F1	TATGTTCTGGAGTACTGGTACGGTATTCAGGAAGACC
	-	reverse	LPS_G25X-R1	AGCATACTTATCGGTGCCAGTCAGACCATGC

Table S3. Mutation primers for LPS_Val26.

mutation point	amino acid substitution	primer name		sequence (5' - 3')
Val26	Ala	forward	LPS_V26A-F1	GCGCTGGAGTACTGGTACGGTATTCAGGAAGACCTG
	Cys		LPS_V26C-F1	TGCCTGGAGTACTGGTACGGTATTCAGGAAGACCTG
	Asp		LPS_V26D-F1	GATCTGGAGTACTGGTACGGTATTCAGGAAGACCTG
	Glu		LPS_V26E-F1	GAACTGGAGTACTGGTACGGTATTCAGGAAGACCTG
	Phe		LPS_V26F-F1	TTCCTGGAGTACTGGTACGGTATTCAGGAAGACCTG
	Gly		LPS_V26G-F1	GGCCTGGAGTACTGGTACGGTATTCAGGAAGACCTG
	His		LPS_V26H-F1	CATCTGGAGTACTGGTACGGTATTCAGGAAGACCTG
	Ile		LPS_V26I-F1	ATTCTGGAGTACTGGTACGGTATTCAGGAAGACCTG
	Lys		LPS_V26K-F1	AAACTGGAGTACTGGTACGGTATTCAGGAAGACCTG
	Leu		LPS_V26L-F1	CTGCTGGAGTACTGGTACGGTATTCAGGAAGACCTG
	Met		LPS_V26M-F1	ATGCTGGAGTACTGGTACGGTATTCAGGAAGACCTG
	Asn		LPS_V26N-F1	AACCTGGAGTACTGGTACGGTATTCAGGAAGACCTG
	Pro		LPS_V26P-F1	CCGCTGGAGTACTGGTACGGTATTCAGGAAGACCTG
	Gln		LPS_V26Q-F1	CAGCTGGAGTACTGGTACGGTATTCAGGAAGACCTG
	Arg		LPS_V26R-F1	CGTCTGGAGTACTGGTACGGTATTCAGGAAGACCTG
	Ser		LPS_V26S-F1	AGCCTGGAGTACTGGTACGGTATTCAGGAAGACCTG
	Thr		LPS_V26T-F1	ACCCTGGAGTACTGGTACGGTATTCAGGAAGACCTG
	Val		LPS_V26V-F1	GTGCTGGAGTACTGGTACGGTATTCAGGAAGACCTG
	Trp		LPS_V26W-F1	TGGCTGGAGTACTGGTACGGTATTCAGGAAGACCTG
	Tyr		LPS_V26Y-F1	TATCTGGAGTACTGGTACGGTATTCAGGAAGACCTG
	-	reverse	LPS_V26X-R1	ACCAGCATACTTATCGGTGCCAGTCAGACC

Table S4. Mutation primers for LPS_Leu27.

mutation point	amino acid substitution	primer name		sequence (5' - 3')
Leu27	Ala	forward	LPS_L27A-F1	GCGGAGTACTGGTACGGTATTCAGGAAGACCTGCAG
	Cys		LPS_L27C-F1	TGCGAGTACTGGTACGGTATTCAGGAAGACCTGCAG
	Asp		LPS_L27D-F1	GATGAGTACTGGTACGGTATTCAGGAAGACCTGCAG
	Glu		LPS_L27E-F1	GAAGAGTACTGGTACGGTATTCAGGAAGACCTGCAG
	Phe		LPS_L27F-F1	TTCGAGTACTGGTACGGTATTCAGGAAGACCTGCAG
	Gly		LPS_L27G-F1	GGCGAGTACTGGTACGGTATTCAGGAAGACCTGCAG
	His		LPS_L27H-F1	CATGAGTACTGGTACGGTATTCAGGAAGACCTGCAG
	Ile		LPS_L27I-F1	ATTGAGTACTGGTACGGTATTCAGGAAGACCTGCAG
	Lys		LPS_L27K-F1	AAAGAGTACTGGTACGGTATTCAGGAAGACCTGCAG
	Leu		LPS_L27L-F1	CTGGAGTACTGGTACGGTATTCAGGAAGACCTGCAG
	Met		LPS_L27M-F1	ATGGAGTACTGGTACGGTATTCAGGAAGACCTGCAG
	Asn		LPS_L27N-F1	AACGAGTACTGGTACGGTATTCAGGAAGACCTGCAG
	Pro		LPS_L27P-F1	CCGGAGTACTGGTACGGTATTCAGGAAGACCTGCAG
	Gln		LPS_L27Q-F1	CAGGAGTACTGGTACGGTATTCAGGAAGACCTGCAG
	Arg		LPS_L27R-F1	CGTGAGTACTGGTACGGTATTCAGGAAGACCTGCAG
	Ser		LPS_L27S-F1	AGCGAGTACTGGTACGGTATTCAGGAAGACCTGCAG
	Thr		LPS_L27T-F1	ACCGAGTACTGGTACGGTATTCAGGAAGACCTGCAG
	Val		LPS_L27V-F1	GTGGAGTACTGGTACGGTATTCAGGAAGACCTGCAG
	Trp		LPS_L27W-F1	TGGGAGTACTGGTACGGTATTCAGGAAGACCTGCAG
	Tyr		LPS_L27Y-F1	TATGAGTACTGGTACGGTATTCAGGAAGACCTGCAG
	-	reverse	LPS_L27X-R1	AACACCAGCATACTTATCGGTGCCAGTCAGAC

Table S5. Mutation primers for LPS_Pro233.

mutation point	amino acid substitution		primer name	sequence (5' - 3')
Pro233	Ala	forward	LPS_P233A-F1	GCGCTGGTGGATCCGGCAAACGCACTG
	Cys		LPS_P233C-F1	TGCCTGGTGGATCCGGCAAACGCACTG
	Asp		LPS_P233D-F1	GATCTGGTGGATCCGGCAAACGCACTG
	Glu		LPS_P233E-F1	GAACTGGTGGATCCGGCAAACGCACTG
	Phe		LPS_P233F-F1	TTCCTGGTGGATCCGGCAAACGCACTG
	Gly		LPS_P233G-F1	GGCCTGGTGGATCCGGCAAACGCACTG
	His		LPS_P233H-F1	CATCTGGTGGATCCGGCAAACGCACTG
	Ile		LPS_P233I-F1	ATTCTGGTGGATCCGGCAAACGCACTG
	Lys		LPS_P233K-F1	AAACTGGTGGATCCGGCAAACGCACTG
	Leu		LPS_P233L-F1	CTGCTGGTGGATCCGGCAAACGCACTG
	Met		LPS_P233M-F1	ATGCTGGTGGATCCGGCAAACGCACTG
	Asn		LPS_P233N-F1	AACCTGGTGGATCCGGCAAACGCACTG
	Pro		LPS_P233P-F1	CCGCTGGTGGATCCGGCAAACGCACTG
	Gln		LPS_P233Q-F1	CAGCTGGTGGATCCGGCAAACGCACTG
	Arg		LPS_P233R-F1	CGTCTGGTGGATCCGGCAAACGCACTG
	Ser		LPS_P233S-F1	AGCCTGGTGGATCCGGCAAACGCACTG
	Thr		LPS_P233T-F1	ACCCTGGTGGATCCGGCAAACGCACTG
	Val		LPS_P233V-F1	GTGCTGGTGGATCCGGCAAACGCACTG
	Trp		LPS_P233W-F1	TGGCTGGTGGATCCGGCAAACGCACTG
	Tyr		LPS_P233Y-F1	TATCTGGTGGATCCGGCAAACGCACTG
	-	reverse	LPS_P233X-R1	GATAGTAGAGGTATCAGTCGCACCAGTAACACCG

Table S6. Mutation primers for LPS_Leu234.

mutation point	amino acid substitution		primer name	sequence (5' - 3')
Leu234	Ala	forward	LPS_L234A-F1	GCGGTGGATCCGGCAAACGCACTGGAC
	Cys		LPS_L234C-F1	TGCGTGGATCCGGCAAACGCACTGGAC
	Asp		LPS_L234D-F1	GATGTGGATCCGGCAAACGCACTGGAC
	Glu		LPS_L234E-F1	GAAGTGGATCCGGCAAACGCACTGGAC
	Phe		LPS_L234F-F1	TTCGTGGATCCGGCAAACGCACTGGAC
	Gly		LPS_L234G-F1	GGCGTGGATCCGGCAAACGCACTGGAC
	His		LPS_L234H-F1	CATGTGGATCCGGCAAACGCACTGGAC
	Ile		LPS_L234I-F1	ATTGTGGATCCGGCAAACGCACTGGAC
	Lys		LPS_L234K-F1	AAAGTGGATCCGGCAAACGCACTGGAC
	Leu		LPS_L234L-F1	CTGGTGGATCCGGCAAACGCACTGGAC
	Met		LPS_L234M-F1	ATGGTGGATCCGGCAAACGCACTGGAC
	Asn		LPS_L234N-F1	AACGTGGATCCGGCAAACGCACTGGAC
	Pro		LPS_L234P-F1	CCGGTGGATCCGGCAAACGCACTGGAC
	Gln		LPS_L234Q-F1	CAGGTGGATCCGGCAAACGCACTGGAC
	Arg		LPS_L234R-F1	CGTGTGGATCCGGCAAACGCACTGGAC
	Ser		LPS_L234S-F1	AGCGTGGATCCGGCAAACGCACTGGAC
	Thr		LPS_L234T-F1	ACCGTGGATCCGGCAAACGCACTGGAC
	Val		LPS_L234V-F1	GTGGTGGATCCGGCAAACGCACTGGAC
	Trp		LPS_L234W-F1	TGGGTGGATCCGGCAAACGCACTGGAC
	Tyr		LPS_L234Y-F1	TATGTGGATCCGGCAAACGCACTGGAC
	-	reverse	LPS_L234X-R1	CGGGATAGTAGAGGTATCAGTCGCACCAGTAAC

Table S7. Mutation primers for LPS_Val235.

mutation point	amino acid substitution	primer name		sequence (5' - 3')
Val235	Ala	forward	LPS_V235A-F1	GCGGATCCGGCAAACGCACTGGACCC
	Cys		LPS_V235C-F1	TGCGATCCGGCAAACGCACTGGACCC
	Asp		LPS_V235D-F1	GATGATCCGGCAAACGCACTGGACCC
	Glu		LPS_V235E-F1	GAAGATCCGGCAAACGCACTGGACCC
	Phe		LPS_V235F-F1	TTCGATCCGGCAAACGCACTGGACCC
	Gly		LPS_V235G-F1	GGCGATCCGGCAAACGCACTGGACCC
	His		LPS_V235H-F1	CATGATCCGGCAAACGCACTGGACCC
	Ile		LPS_V235I-F1	ATTGATCCGGCAAACGCACTGGACCC
	Lys		LPS_V235K-F1	AAAGATCCGGCAAACGCACTGGACCC
	Leu		LPS_V235L-F1	CTGGATCCGGCAAACGCACTGGACCC
	Met		LPS_V235M-F1	ATGGATCCGGCAAACGCACTGGACCC
	Asn		LPS_V235N-F1	AACGATCCGGCAAACGCACTGGACCC
	Pro		LPS_V235P-F1	CCGGATCCGGCAAACGCACTGGACCC
	Gln		LPS_V235Q-F1	CAGGATCCGGCAAACGCACTGGACCC
	Arg		LPS_V235R-F1	CGTGATCCGGCAAACGCACTGGACCC
	Ser		LPS_V235S-F1	AGCGATCCGGCAAACGCACTGGACCC
	Thr		LPS_V235T-F1	ACCGATCCGGCAAACGCACTGGACCC
	Val		LPS_V235V-F1	GTGGATCCGGCAAACGCACTGGACCC
	Trp		LPS_V235W-F1	TGGGATCCGGCAAACGCACTGGACCC
	Tyr		LPS_V235Y-F1	TATGATCCGGCAAACGCACTGGACCC
	-	reverse	LPS_V235X-R1	CAGCGGGATAGTAGAGGTATCAGTCGCACC

Table S8. Top 20 ranking predicted to fit the rule of "high improvement".[a]

		categories of prediction	
ranking	P233X/L234X/V235X	medium	high
1	DPE	0.027	0.973
2	EPE	0.027	0.973
3	DPG	0.030	0.970
4	EPG	0.031	0.969
5	DPD	0.031	0.969
6	DPN	0.031	0.969
7	EPD	0.032	0.968
8	EPN	0.032	0.968
9	DGE	0.036	0.964
10	EGE	0.036	0.964
11	DPS	0.040	0.960
12	DGG	0.040	0.960
13	EPS	0.040	0.960
14	DNE	0.040	0.960
15	EGG	0.041	0.959
16	ENE	0.041	0.959
17	DKE	0.041	0.959
18	DGD	0.041	0.959
19	DGN	0.042	0.958
20	DQE	0.042	0.958

[a] The numbers in categories of prediction designate probabilities for category prediction.

Table S9. 20 ranking predicted to fit the rule of "medium improvement".[a]

		categories of prediction	
ranking	P233X/L234X/V235X	medium	high
2637	MWG	0.445	0.555
2638	HQA	0.445	0.555
2639	ADT	0.445	0.555
2640	ISN	0.445	0.555
2641	MNA	0.445	0.555
2642	HFN	0.445	0.555
2643	VCE	0.446	0.554
2644	HKK	0.446	0.554
2645	QYM	0.446	0.554
2646	HWN	0.446	0.554
2647	HES	0.446	0.554
2648	AAR	0.446	0.554
2649	NEL	0.447	0.553
2650	HMD	0.447	0.553
2651	MMG	0.447	0.553
2652	QVM	0.447	0.553
2653	AET	0.448	0.552
2654	NSY	0.448	0.552
2655	KNE	0.449	0.551
2656	HQK	0.449	0.551

[a] The numbers in categories of prediction designate probabilities for category prediction.

Table S10. Mutation primers for high 20 mutants.

prediction ranking	strain		primer name	sequence (5' - 3')
1	LPS_P233D/L234P/V235E	forward	LPS_DPE-F1	GATCCGGAAGATCCGGCAAACGCACTGGACC
2	LPS_P233E/L234P/V235E		LPS_EPE-F1	GAACCGGAAGATCCGGCAAACGCACTGGACC
3	LPS_P233D/L234P/V235G		LPS_DPG-F1	GATCCGGGCGATCCGGCAAACGCACTGGACC
4	LPS_P233E/L234P/V235G		LPS_EPG-F1	GAACCGGGCGATCCGGCAAACGCACTGGACC
5	LPS_P233D/L234P/V235D		LPS_DPD-F1	GATCCGGATGATCCGGCAAACGCACTGGACC
6	LPS_P233D/L234P/V235N		LPS_DPN-F1	GATCCGAACGATCCGGCAAACGCACTGGACC
7	LPS_P233E/L234P/V235D		LPS_EPD-F1	GAACCGGATGATCCGGCAAACGCACTGGACC
8	LPS_P233E/L234P/V235N		LPS_EPN-F1	GAACCGAACGATCCGGCAAACGCACTGGACC
9	LPS_P233D/L234G/V235E		LPS_DGE-F1	GATGGCGAAGATCCGGCAAACGCACTGGACC
10	LPS_P233E/L234G/V235E		LPS_EGE-F1	GAAGGCGAAGATCCGGCAAACGCACTGGACC
11	LPS_P233D/L234P/V235S		LPS_DPS-F1	GATCCGAGCGATCCGGCAAACGCACTGGACC
12	LPS_P233D/L234G/V235G		LPS_DGG-F1	GATGGCGGCGATCCGGCAAACGCACTGGACC
13	LPS_P233E/L234P/V235S		LPS_EPS-F1	GAACCGAGCGATCCGGCAAACGCACTGGACC
14	LPS_P233D/L234N/V235E		LPS_DNE-F1	GATAACGAAGATCCGGCAAACGCACTGGACC
15	LPS_P233E/L234G/V235G		LPS_EGG-F1	GAAGGCGGCGATCCGGCAAACGCACTGGACC
16	LPS_P233E/L234N/V235E		LPS_ENE-F1	GAAAACGAAGATCCGGCAAACGCACTGGACC
17	LPS_P233D/L234K/V235E		LPS_DKE-F1	GATAAAGAAGATCCGGCAAACGCACTGGACC
18	LPS_P233D/L234G/V235D		LPS_DGD-F1	GATGGCGATGATCCGGCAAACGCACTGGACC
19	LPS_P233D/L234G/V235N		LPS_DGN-F1	GATGGCAACGATCCGGCAAACGCACTGGACC
20	LPS_P233D/L234Q/V235E		LPS_DQE-F1	GATCAGGAAGATCCGGCAAACGCACTGGACC
-	-	reverse	LPS-L7-R1	GATAGTAGAGGTATCAGTCGCACCAGTAAC

Table S11. Mutation primers for medium 20 mutants.

prediction ranking	strain		primer name	sequence (5' - 3')
2637	LPS_P233M/L234W/V235G	forward	LPS_MWG-F1	ATGTGGGGCGATCCGGCAAACGCACTGGACC
2638	LPS_P233H/L234Q/V235A		LPS_HQA-F1	CATCAGGCGGATCCGGCAAACGCACTGGACC
2639	LPS_P233A/L234D/V235T		LPS_ADT-F1	GCGGATACCGATCCGGCAAACGCACTGGACC
2640	LPS_P233I/L234S/V235N		LPS_ISN-F1	ATTAGCAACGATCCGGCAAACGCACTGGACC
2641	LPS_P233M/L234N/V235A		LPS_MNA-F1	ATGAACGCGGATCCGGCAAACGCACTGGACC
2642	LPS_P233H/L234F/V235N		LPS_HFN-F1	CATTTCAACGATCCGGCAAACGCACTGGACC
2643	LPS_P233V/L234C/V235E		LPS_VCE-F1	GTGTGCGAAGATCCGGCAAACGCACTGGACC
2644	LPS_P233H/L234K/V235K		LPS_HKK-F1	CATAAAAAAGATCCGGCAAACGCACTGGACC
2645	LPS_P233Q/L234Y/V235M		LPS_QYM-F1	CAGTATATGGATCCGGCAAACGCACTGGACC
2646	LPS_P233H/L234W/V235N		LPS_HWN-F1	CATTGGAACGATCCGGCAAACGCACTGGACC
2647	LPS_P233H/L234E/V235S		LPS_HES-F1	CATGAAAGCGATCCGGCAAACGCACTGGACC
2648	LPS_P233A/L234A/V235R		LPS_AAR-F1	GCGGCGCGTGATCCGGCAAACGCACTGGACC
2649	LPS_P233N/L234E/V235L		LPS_NEL-F1	AACGAACTGGATCCGGCAAACGCACTGGACC
2650	LPS_P233H/L234M/V235D		LPS_HMD-F1	CATATGGATGATCCGGCAAACGCACTGGACC
2651	LPS_P233M/L234M/V235G		LPS_MMG-F1	ATGATGGGCGATCCGGCAAACGCACTGGACC
2652	LPS_P233Q/L234V/V235M		LPS_QVM-F1	CAGGTGATGGATCCGGCAAACGCACTGGACC
2653	LPS_P233A/L234E/V235T		LPS_AET-F1	GCGGAAACCGATCCGGCAAACGCACTGGACC
2654	LPS_P233N/L234S/V235Y		LPS_NSY-F1	AACAGCTATGATCCGGCAAACGCACTGGACC
2655	LPS_P233K/L234N/V235E		LPS_KNE-F1	AAAAACGAAGATCCGGCAAACGCACTGGACC
2656	LPS_P233H/L234Q/V235K		LPS_HQK-F1	CATCAGAAAGATCCGGCAAACGCACTGGACC
-	-	reverse	LPS-L7-R1	GATAGTAGAGGTATCAGTCGCACCAGTAAC

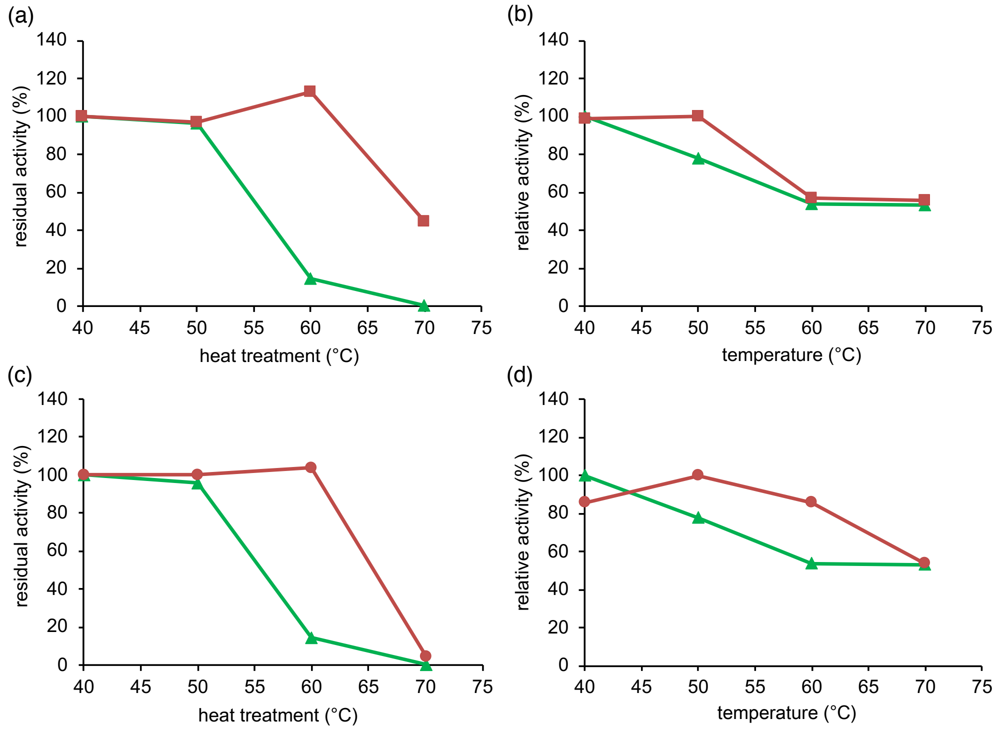

Figure S1. (a) Thermostability and (b) optimum temperature of the P233G/L234E/V235M mutant and (c) thermostability and (d) optimum temperature of the P233H/L234V/V235H mutant. In (a) and (c), horizontal axis: temperature of heat treatment for 30 min, vertical axis: relative activity after heat treatment. In (b) and (d), horizontal axis: assay temperature, vertical axis: relative enzymatic activity. ▲: LPS_wild-type, ■: P233G/L234E/V235M, ●: P233H/L234V/V235H.

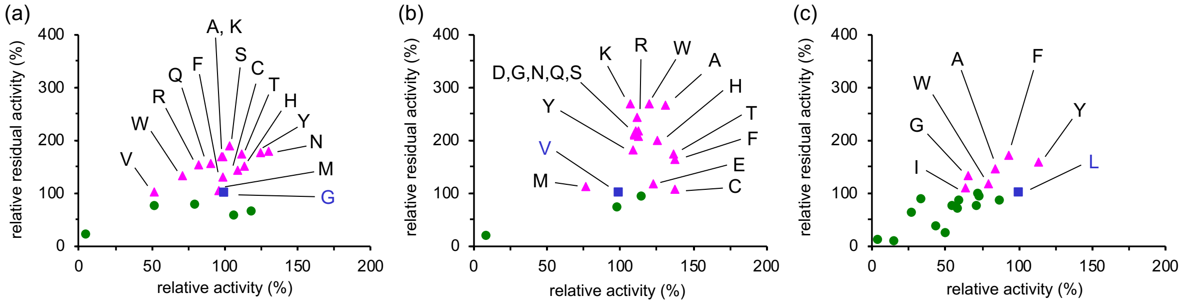

Figure S2. Thermostability plots for (a) G25X, (b) V26X, and (c) L27X single mutants using relative activity without heat treatment (horizontal axis) and relative residual activity after heat treatment at 60 °C for 30 min (vertical axis). The blue square represents the wild-type enzyme while the pink triangle represents the mutants with improved thermostability, and the green circle represents the mutants with reduced thermostability.

AAindex No.	name	A	C	D	E	F	G	H	I	K	L	M	N	P	Q	R	S	T	V	W	Y
401	Isoelectric point (Zimmerman et al., 1968)	6	5	3	3	5	2	8	6	10	6	6	5	6	6	11	6	6	6	6	6
80	Normalized van der Waals volume (Fauchere et al., 1988)	1	2	3	4	6	2	5	4	5	4	4	3	3	4	6	2	3	3	8	6
99	Alpha-helix indices for beta-proteins (Geisow-Roberts, 1980)	2	1	2	2	0	2	1	1	1	1	1	1	0	1	0	1	1	1	2	1
102	Beta-strand indices for beta-proteins (Geisow-Roberts, 1980)	1	1	1	0	1	2	1	1	1	1	1	1	1	1	1	1	1	1	1	1
444	Side-chain contribution to protein stability (kJ/mol) (Takano-Yutani, 2001)	10	3	5	4	23	2	12	17	11	17	12	4	15	2	7	3	7	15	24	17
510	The stability scale from the knowledge-based atom-atom potential (Zhou-Zhou, 2004)	2	4	2	2	6	2	3	5	2	5	4	2	2	2	3	2	2	4	6	5
151	Hydropathy index (Kyte-Doolittle, 1982)	2	3	-4	-4	3	2	-3	5	-4	4	2	-4	-2	-4	-5	-1	-1	4	-1	-1
62	Normalized frequency of turn (Crawford et al., 1973)	1	1	1	1	1	2	1	1	1	1	1	1	1	1	1	1	1	0	1	1
432	Free energy in beta-strand region (Munoz-Serrano, 1994)	1	1	1	1	1	2	1	1	1	1	1	1	3	1	1	1	1	0	1	1
430	Free energy in alpha-helical region (Munoz-Serrano, 1994)	1	1	1	1	1	2	1	1	1	1	1	1	2	1	1	1	1	1	1	1
400	Polarity (Zimmerman et al., 1968)	0	1	50	50	0	2	52	0	50	0	1	3	2	4	52	2	2	0	2	2
148	Side chain interaction parameter (Krigbaum-Komoriya, 1979)	4	2	6	6	3	2	6	2	8	4	2	6	7	6	7	5	5	3	3	4
136	Amino acid distribution (Jukes et al., 1975)	5	1	4	3	2	2	1	3	4	5	1	3	3	2	3	5	4	4	1	2

Figure S3. AAindex.
